# Supplementary material for: A bibliometric analysis of the gender gap in the authorship of leading medical journals
Source: Commun Med (Lond). 2023 Dec 11;3:179. doi: 10.1038/s43856-023-00417-3 (PMC10713825; doi:10.1038/s43856-023-00417-3)
Supplement: Supplementary file 2 — Supplementary Information [file 43856_2023_417_MOESM2_ESM.pdf]

A Bibliometric Analysis of the Gender Gap in the Authorship of Leading Medical Journals

Supplementary Information

Oscar Brück[1]

1 *Hematoscope Lab, Comprehensive Cancer Center & Center of Diagnostics, Helsinki University Hospital, Helsinki, Finland & Department of Oncology, University of Helsinki, Helsinki, Finland*

Supplementary table 1. Linear regression models with log10-transformed year-averaged citation count.

Model	Model covariates	Covariate	Coefficient estimate	R^2	P-value
1	Cov1	Cov1	0.0012, p<0.001	0.026	p<0.001
2	Cov2	Cov2	0.099, p<0.001	0.0061	p<0.001
3	Cov3	Cov3	0.083, p<0.001	0.035	p<0.001
4	Cov1+ Cov2+Cov1*Cov2	Cov1	0.0030, p<0.001	0.036	p<0.001
4	Cov1+ Cov2+Cov1*Cov2	Cov2	0.12, p<0.001	0.036	p<0.001
4	Cov1+ Cov2+Cov1*Cov2	Cov1*Cov2	-0.0018, p<0.001	0.036	p<0.001
5	Cov1+ Cov3+Cov1*Cov3	Cov1	0.0011, p<0.001	0.034	p<0.001
5	Cov1+ Cov3+Cov1*Cov3	Cov3	0.068, p<0.001	0.034	p<0.001
5	Cov1+ Cov3+Cov1*Cov3	Cov1*Cov3	0.00073, p<0.001	0.034	p<0.001

Abbreviations: Cov1, Number of authors; Cov2 = First author gender (men); Cov3 = Last author gender (men); Cov1*Cov2, interaction of Cov1 and Cov2; R^2, adjusted R-squared

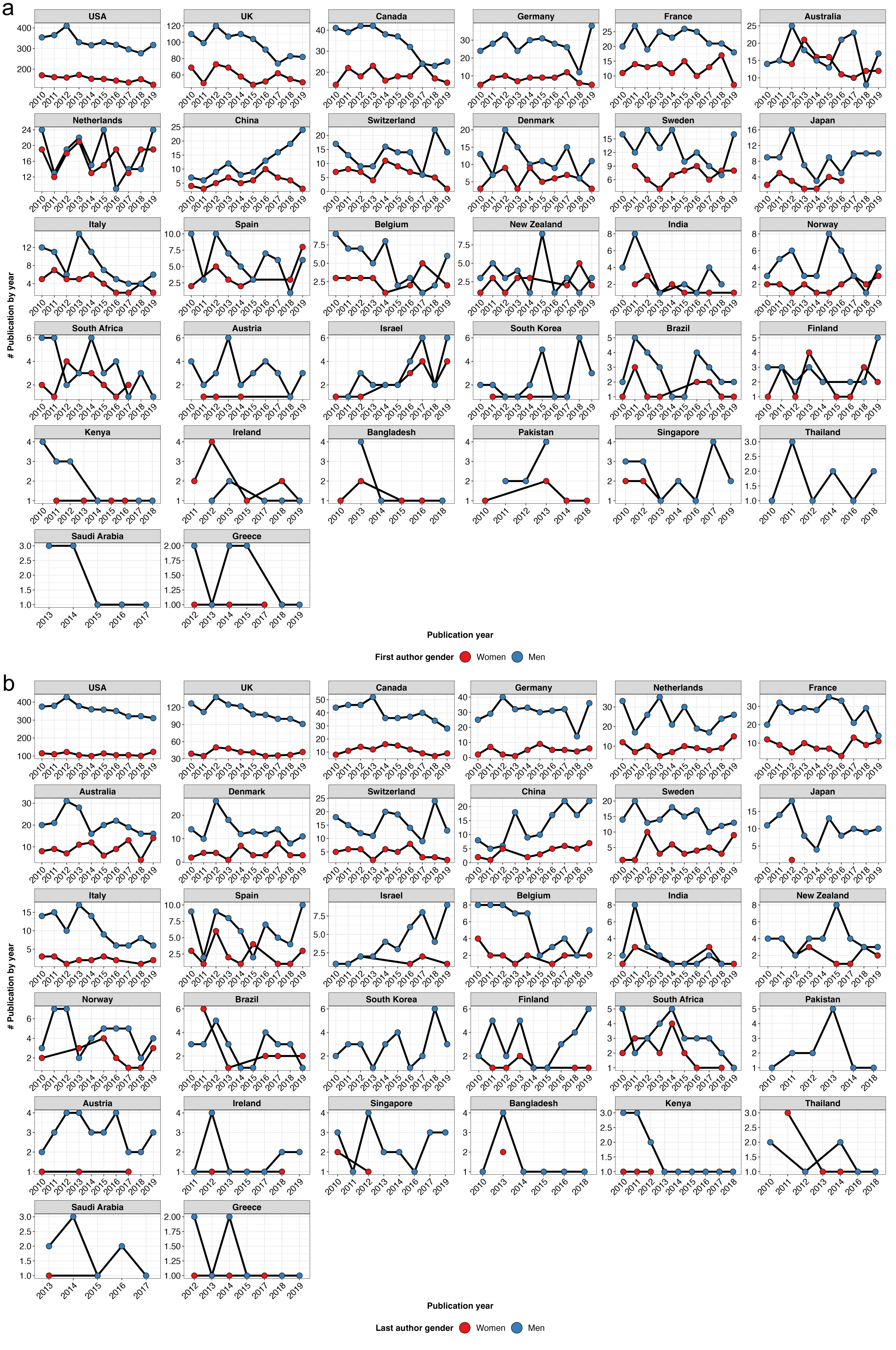

Supplementary Figure 1. Line plot illustrating the number of publications by their publishing year from the top 32 most productive cities based on the gender of the (a) first and (b) last author.

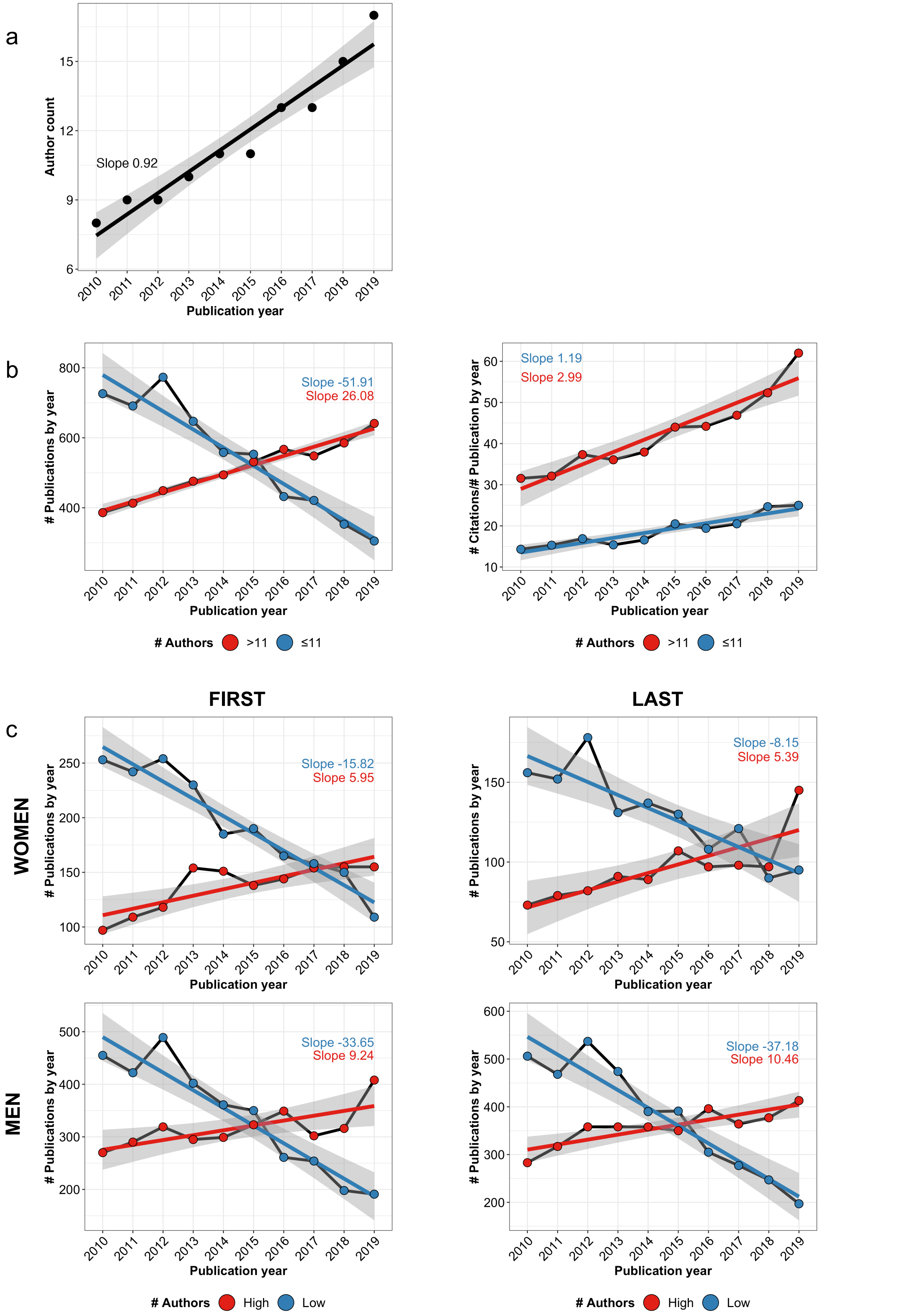

Supplementary Figure 2. (a) Fitted linear regression for the number of authors in publications by their publishing year. (b) Line plot and fitted linear regression for the number of publications (left) and yearly-averaged citations per publication (right) by their publishing year and number of authors. (c) Line plot and fitted linear regression for the number of publications by their publishing year and number of authors. The panel is divided by first and last author gender.
